# Shattered Hearts: Exploring Takotsubo Cardiomyopathy Triggered by a Mechanical Fall in an 88-Year-Old Woman

**DOI:** 10.7759/cureus.82651

**Published:** 2025-04-20

**Authors:** Girma M Ayele, Rediet Tefera Atalay, Kristin Slater, Shahnoza Dusmatova, Bamlak Gebremariam, Ahmed Brgdar, Miriam Michael, Fatima Urooj

**Affiliations:** 1 Internal Medicine, Howard University Hospital, Washington, D.C., USA; 2 Cardiovascular Disease, Howard University Hospital, Washington, D.C., USA

**Keywords:** broken heart syndrome, non ischemic cardiomyopathy, stress cardiomyopathy, takotsubo cardiomyopathy, tts

## Abstract

Takotsubo cardiomyopathy, also known as "Broken Heart Syndrome," manifests when the heart muscle experiences sudden stun or weakening, typically triggered by intense emotional or physical stress. Common triggers include the death of a loved one, a severe accident, a heated argument, an unexpected loss, or a sudden illness, thus earning the syndrome its colloquial name, broken heart syndrome. The symptoms closely resemble those of acute myocardial ischemia. While emotional stress is a well-known trigger, blunt chest trauma has been reported as a trigger in only a few studies. Similar to emotional stress, blunt trauma can also induce a surge in catecholamines, leading to myocardial stunning and transient left ventricular dysfunction. We present a distinctive case of Takotsubo cardiomyopathy following a minor ground-level fall, with no cardiac symptoms.

## Introduction

First identified in Japan in 1990, Takotsubo syndrome (TTS), alternatively referred to as stress cardiomyopathy, broken heart syndrome, or apical ballooning syndrome, has gained recognition as a significant manifestation of acute, reversible myocardial injury. It is distinguished by transient regional systolic dysfunction in the left ventricle (hypokinesis, akinesis, or dyskinesis) [1,2]. The clinical manifestations of Takotsubo cardiomyopathy bear resemblance to those observed in myocardial ischemia following acute plaque rupture. However, distinctive features include regional wall motion abnormalities that extend beyond a singular coronary vascular bed and the absence of epicardial coronary occlusion [1].

The initiation of transient left ventricular apical ballooning syndrome frequently occurs following emotional or physical stress [3]. Typically, Takotsubo syndrome is preceded by intense emotional triggers, although in up to one-third of patients, no identifiable trigger exists. Takotsubo syndrome may also manifest in various medical conditions, including sepsis, neurological disorders, and pheochromocytoma. Additionally, drugs like dopamine, dobutamine, epinephrine, or norepinephrine, particularly in the context of cardiovascular stress tests or anesthesia, can induce Takotsubo syndrome. This condition is termed as Takotsubo phenocopies, which are different from classic Takotsubo syndrome, which is caused by emotional stress [4].

Takotsubo cardiomyopathy accounts for 1-3% of acute coronary syndrome (ACS) cases, with a strong female predominance-diagnosed in 5-6% of women presenting with suspected ACS [5]. Diagnosis is based on clinical history, elevated cardiac biomarkers, ST elevation on ECG, echocardiography, and absence of obstructive coronary disease on angiography. Management is mainly supportive. Recurrence rates range from 4-20% over 10 years, and all-cause mortality can reach 20%, comparable to that of ACS [6,7 ].

## Case presentation

An 88-year-old female patient with a medical history of dementia, type 2 diabetes, hypertension, and hyperlipidemia presented to the Emergency Department following a fall. The patient indicated that she tripped and fell forward, with no loss of consciousness or trauma to other body parts. She denied experiencing chest pain, shortness of breath, cough, fever, nausea/vomiting, numbness, or weakness. The patient was typically active and independent in her daily activities and remained compliant with her medications.

**Figure 1:**
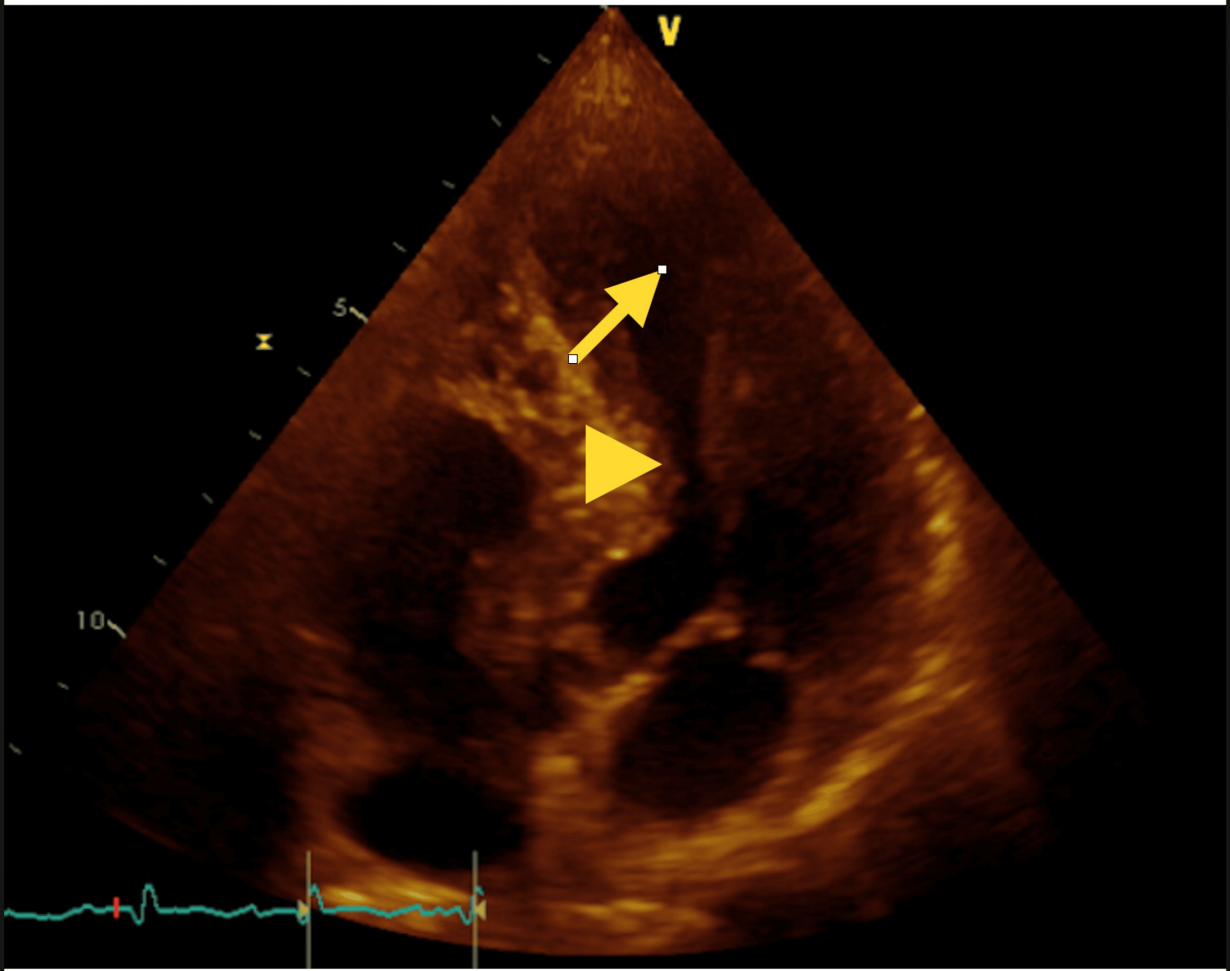
Apical four-chamber view demonstrating classic features of Takotsubo cardiomyopathy, with marked apical ballooning (arrow) and basal hyperkinesis (arrowhead).

**Figure 2:**
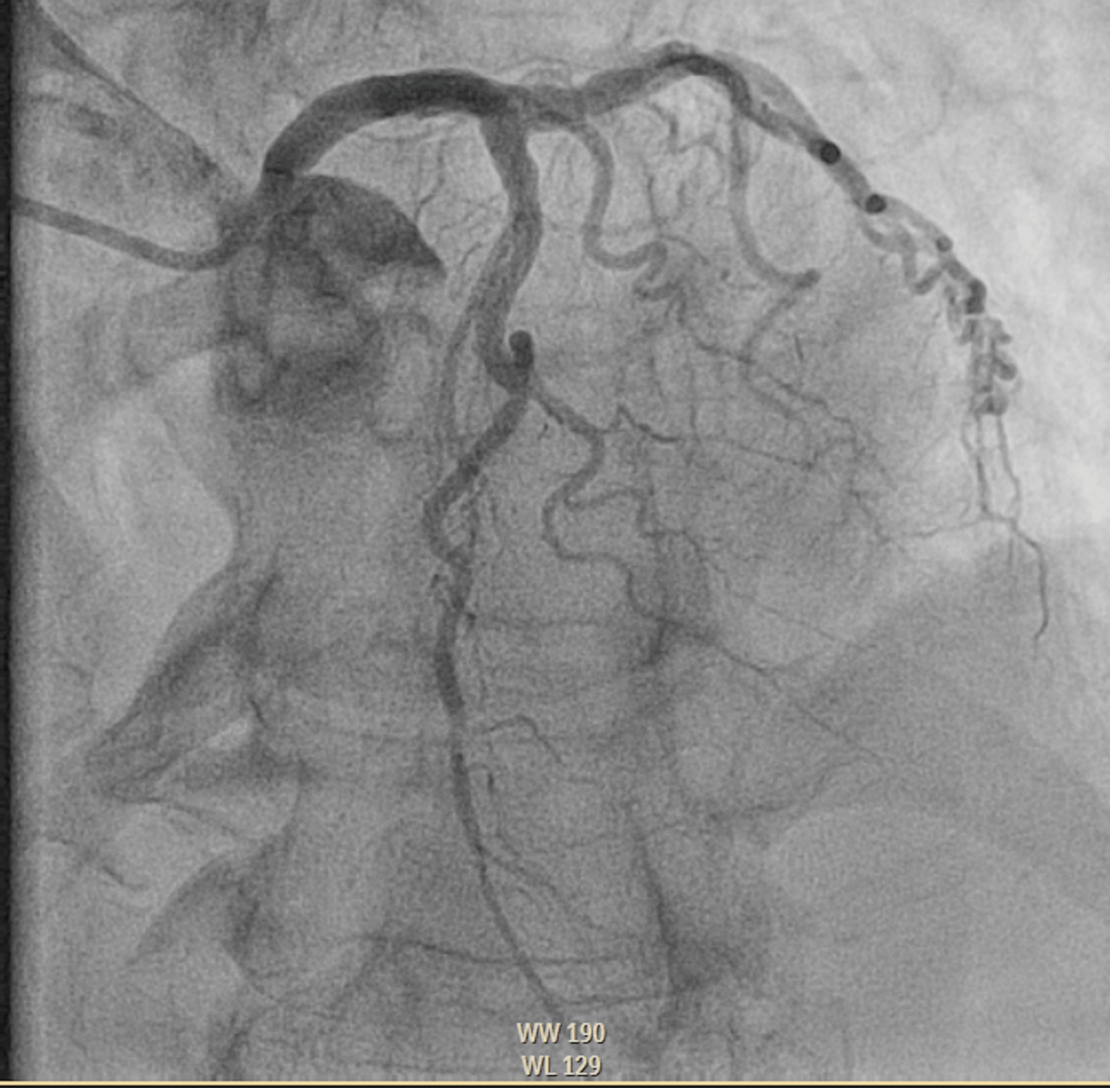
Left anterior oblique (LAO) cranial view from left heart catheterization demonstrating patent coronary arteries with no significant stenosis.

Upon evaluation in the emergency department (ED), the patient was alert and oriented with elevated blood pressure (151/117), tachycardia (104 beats per minute), and saturating >94% on room air (RA). Her exam findings were unremarkable. Trauma imaging revealed no abnormalities, and all laboratory tests were generally normal, except for an elevated troponin level of 0.69 ng/mL, which increased to 3.417 ng/mL within 4 hours. The electrocardiogram (EKG) did not display dynamic ST-T segment changes. Echocardiography (Fig. 1) revealed new left ventricular dysfunction with an ejection fraction (EF) of 40-45%, and regional wall motion abnormality (hyperdynamic basal segment and akinetic mid and distal segments), raising the possibility of Takotsubo cardiomyopathy versus multi-vessel coronary artery disease. The patient was subsequently taken to the catheterization lab for further evaluation for possible non-ST-segment elevation myocardial infarction (NSTEMI). Left heart catheterization results demonstrated mild proximal and distal left anterior descending (LAD) disease (Fig. 2). This ruled out obstructive coronary disease, favoring the diagnosis of Takotsubo cardiomyopathy. The patient was started on losartan and metoprolol. The patient was discharged with outpatient cardiology follow-up. 

## Discussion

Takotsubo cardiomyopathy, colloquially known as broken heart syndrome, is an acute and transient heart condition characterized by severe left ventricular dysfunction frequently precipitated by a stressful event [7]. The term "Takotsubo" originates from Japanese, describing a pot used for trapping octopuses, chosen due to the resemblance of the affected heart's shape to the pot, with a narrow neck and wide base [8]. First described in Japan in 1990 by Dr. Hikaru Sato and colleagues, Takotsubo cardiomyopathy gained recognition for its unique ventricular ballooning pattern on imaging studies [9]. 

The prevalence of Takotsubo cardiomyopathy has been increasingly acknowledged globally, although underdiagnosis and misdiagnosis may lead to its prevalence being underestimated [10]. While initially considered rare, it is now recognized as a significant cause of acute heart failure, particularly among postmenopausal women [7]. Studies suggest that Takotsubo cardiomyopathy may account for up to 2% of all suspected acute coronary syndrome cases, with a higher incidence in women over 50 years old [11]. 

Takotsubo cardiomyopathy is defined as a clinical syndrome involving a brief (<21 days) dysfunction of the left ventricle (LV) associated with emotional or physical stress, usually occurring 1 to 5 days prior. It is identified by LV regional wall motion abnormalities extending beyond a single coronary artery distribution [5]. Numerous diagnostic criteria exist for Takotsubo Syndrome; recently, the International Takotsubo Diagnostic Criteria (InterTAK Diagnostic Criteria) have been proposed [12]. The InterTAK International Registry Group has devised a straightforward scoring system that considers five clinical variables from the patient's history and two variables from the electrocardiogram (ECG). This system generates a score that corresponds to the likelihood of stress cardiomyopathy, known as the InterTAK diagnostic score (Table 1) [13].

**Table 1:** International Takotsubo (InterTAK) diagnostic score Source: Gianni et al. [3]

Criteria	Points
Female	25
Emotional trigger	24
Physical trigger	13
Absence of ST-segment depression	12
Psychiatric disorders	11
Neurologic disorders	9
QTc prolongation	6
Diagnosis (Cutoff Value (Range 0–100))	
≥50	≤31
Takotsubo	Acute coronary syndrome
(Specificity 95%)	(Specificity 95%)

The precise cause of Takotsubo cardiomyopathy remains unknown, but the influence of catecholamines is significant in both primary TTS(triggered by unexpected stress) and secondary TTS (related to physical illness). Numerous clinical pieces of evidence have highlighted catecholamines as a causative factor in triggering left ventricular dysfunction due to stunning in TTS. Other postulated mechanisms include microvascular dysfunction and coronary artery spasm [8]. Studies investigating both epicardial and microvascular spasms have shown that intracoronary adenosine administration improves myocardial function, particularly in cases of microcirculatory dysfunction, although it remains uncertain whether the observed vascular dysfunctions are causal or a consequence of TTS episodes. The transient nature of TTS may represent a protective mechanism against supraphysiological catecholamine levels, preserving energy by downregulating contractile function for LV recovery [11]. 

**Figure 3:**
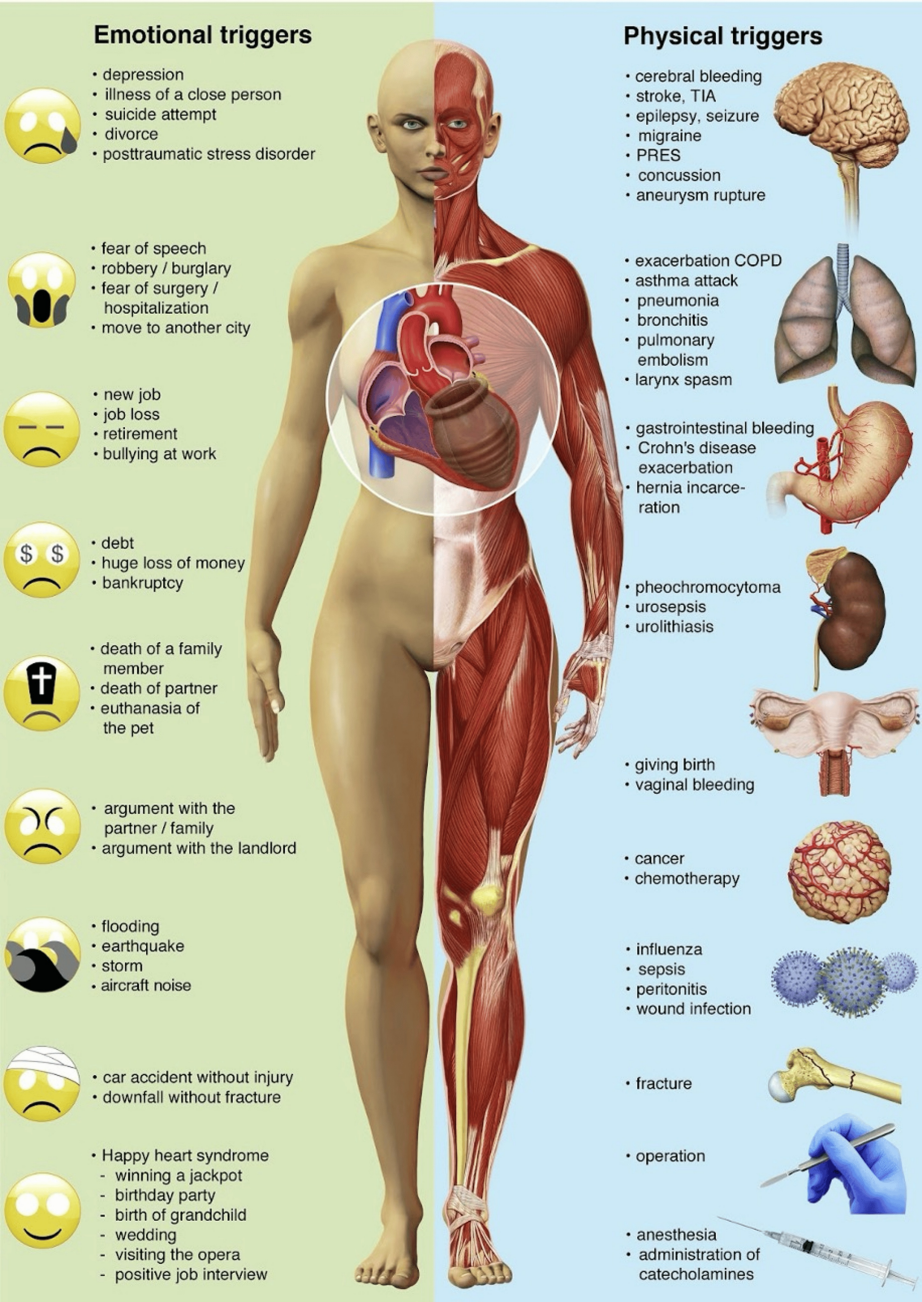
Primary and secondary cause of Takotsubo syndrome Image from Lyon et al. [6], reproduced with the permission of the author, Dr. Alexander Lyon (granted via email on 2/17/2024).

There are different causes of TTS, broadly divided as emotional or physical causes (Fig. 3) [6]. Physical factors were more commonly associated with the onset of TSS than emotional factors, with physical triggers occurring in 36.0% of cases compared to emotional triggers in 27.7%. Additionally, 28.5% of patients presented with no discernible precipitating factor for the syndrome [8]. These findings are further expanded upon in the COUNTS study, which represents the largest series of Takotsubo syndrome patients reported to date. Systematic review revealed a relatively high prevalence of psychological disorders (24%; range: 0-49%), pulmonary diseases (15%; range: 0-22%), malignancies (10%; range: 4-29%), neurologic diseases (7%; range: 0-22%), chronic kidney disease (7%; range: 2-27%), and thyroid diseases (6%; range: 0-37%) among patients with Takotsubo syndrome [14]. Among psychological factors, drug abuse, anxiety disorders, and mood disorders have been identified as potential triggers for TSS [15]. Furthermore, postmenopausal patients with TTS have been shown to exhibit abnormal vasoreactivity and a symptomatic response to acute mental stress [16].

There are different patterns of transient hypokinesis observed on echocardiogram, with no notable difference in clinical presentation, and the underlying reason remains unclear. The most common pattern is apical ballooning, characterized by the involvement of the left ventricular wall with a hyperdynamic base, as observed in our patient. The second most common pattern shows hypokinesis of the mid-ventricle with relative sparing of the apex. Another pattern, known as inverted or reverse Takotsubo, presents with basal hypokinesis, sparing the mid-ventricle and apex. A rare variant involves dysfunction of an isolated segment, typically the anterolateral segment. The final pattern is global hypokinesis, characterized by widespread left ventricular dysfunction [17].

The treatment is primarily supportive. While beta blockers and Renin-Angiotensin-Aldosterone System (RAAS) inhibitors have been suggested, their initiation has not shown clear benefits in patients with rapid recovery. Repeat imaging is recommended within four days of the initial diagnosis, with approximately 50-70% of patients showing rapid improvement. However, a slower recovery has been associated with a worse long-term prognosis. Recurrence is rare, occurring in approximately 3-4% of cases [18].

## Conclusions

In conclusion, this case emphasizes the importance of considering Takotsubo cardiomyopathy in patients presenting with trauma, even when typical cardiac symptoms are absent. While it is commonly associated with emotional or physical stress, this case illustrates that even minor physical trauma, particularly chest trauma in postmenopausal women, can trigger this condition. Clinicians should remain vigilant in recognizing the possibility of Takotsubo cardiomyopathy in such cases, especially in the presence of heart failure, EKG changes, or elevated biomarkers, to ensure timely diagnosis and appropriate management.

Is there a way to prevent the occurrence of Takotsubo cardiomyopathy, given the known triggers? This is a question that future studies may help to answer.
